# Risk factors and impact of hypertriglyceridemia in preterm infants under 32 weeks of gestation: optimizing intravenous lipid emulsion infusion rates–a single center retrospective study

**DOI:** 10.3389/fped.2025.1520420

**Published:** 2025-06-11

**Authors:** Yoong-A Suh, Ji Hyun Park, Bumhee Park, Seoheui Choi, Moon Sung Park, Jang Hoon Lee

**Affiliations:** ^1^ Department of Pediatrics, Ajou University Hospital, Ajou University School of Medicine, Suwon, Republic of Korea; ^2^ Office of Biostatistics, Medical Research Collaborating Center, Ajou Research Institute for Innovative Medicine, Ajou University Medical Center, Suwon, Republic of Korea

**Keywords:** intravenous fat emulsions, parenteral nutrition, hypertriglyceridemia, premature infants, retinopathy of prematurity

## Abstract

**Introduction:**

Intravenous lipid emulsions are essential for parenteral nutrition in preterm infants. However, hypertriglyceridemia (HTG) is a common complication that can lead to severe complications if left untreated. This study analyzed factors influencing HTG and the effects of reduced intravenous lipid emulsion dosages.

**Methods:**

A retrospective cohort study involving premature infants born at less than 32 weeks of gestation and admitted to Ajou University Hospital between 2017 and 2022 was conducted. Infants with documented triglyceride levels within the first 10 days of life were included. The risk factors for HTG and the time to normalize triglyceride levels after intravenous lipid dose reduction were evaluated.

**Results:**

HTG was diagnosed in 104 infants. Significant risk factors included lower birth weight (OR = 0.75; 95% CI, 0.66–0.85; *p* < 0.001), treated for patent ductus arteriosus (OR = 2.47; 95% CI, 1.30–4.69; *p* = 0.006), intravenous lipid emulsion intake (OR = 1.79; 95% CI, 1.25–2.55; *p* = 0.001), and serum glucose level (OR = 1.01; 95% CI, 1.00–1.02; *p* = 0.023). The average triglyceride level in the HTG group was 287.8 mg/dl, with intravenous lipid doses of 2.7 g/kg/day. Reducing intravenous lipid doses by 0.27 ± 0.46 g/kg/day shortened the triglyceride normalization time to 4.9 ± 5.0 days and reduced the risk of retinopathy of prematurity stage 2 or higher (OR = 0.59; 95% CI, 0.40–0.88; *p* = 0.011). The fat overload infusion rate, which induces HTG, was 0.332 cc/kg/h (1.6 g/kg/day).

**Discussion:**

Lower birth weight, treated patent ductus arteriosus, serum glucose levels, and higher intravenous lipid levels are significant risk factors for HTG. Adjusting the lipid dose aids in triglyceride normalization and reducing severe retinopathy of prematurity risk. Careful monitoring and management of intravenous lipid emulsion infusion rates are crucial to prevent HTG.

## 1 Introduction

Intravenous lipid emulsions (ILE) play an important role in the parenteral nutrition (PN) of preterm infants by providing a concentrated source of essential fatty acids and energy (1, 2). Preterm infants often require higher doses of ILE than adults do to meet their growth, development, and daily metabolic needs (3). Infants who receive parenteral nutrition often experience a metabolic complication known as hypertriglyceridemia (HTG) (1, 3, 4). If left untreated, patients with HTG may develop complications, such as pancreatitis and lipid pneumonitis, and neurological changes, such as kernicterus (3, 5). In the past, neonatologists initiated treatment with conservative doses of macronutrients, including amino acids (AA) at 0.5 g/kg/day and ILE at 0.5 g/kg/day, as very low birth weight (VLBW) infants were believed to have limited metabolic tolerance for higher doses (6, 7). Following European Society for Pediatric Gastroenterology, Hepatology, and Nutrition (ESPGHAN) and American Society of Parenteral and Enteral Nutrition (ASPEN) guidelines, our current neonatal nutrition protocol initiates AA at 2.0 g/kg/day (2, 3, 8). In preterm infants with HTG, the ILE dose is typically reduced to 0.5–1.5 g/kg/day (3, 9). However, a subsequent reduction in ILE intake can lead to a decrease in overall energy intake, resulting in growth retardation, especially in VLBW infants (6). Another complication to consider when administering ILEs is fat overload syndrome (3, 10, 11). Fat overload syndrome manifests as hepatosplenomegaly, respiratory distress, jaundice, and spontaneous hemorrhage (3, 12). The side effects associated with fat overload syndrome are caused by elevated serum triglyceride (TG) levels, which occur when the rate of infusion exceeds the rate of lipid hydrolysis (3). Despite these side effects of HTG, there remains a lack of precise guidelines for adjusting lipid infusion rates based on TG, and there is a scarcity of studies analyzing the impact of HTG on preterm infants. ASPEN recommends the maximum rate for ILE is 0.15 g/kg/h for soybean oil, it depends on the ILE types (3). Regarding the 20% SMOFLipid® (Fresenius Kabi, Uppsala, Sweden) formula used in our hospital, the domestic approval requirements are as follows: ILE was gradually increased from 0.5 to 1.0 g/kg/day as daily fat to 3.0 g/kg/day, doses exceeding this are not recommended. The administration rate should not exceed 0.125 g/kg/h of fat (2). In the management of VLBW infants, it is crucial to provide adequate caloric intake through PN while minimizing complications that can arise from the use of ILE. Therefore, understanding the risk factors and significance of HTG is essential to ensure optimal care of these vulnerable infants.

The primary objective of this study is to analyze neonatal factors and time to normalize TG levels and to determine the effect on infants when ILE was reduced by 0–2 g/kg/day in the group with HTG levels, in single center study. In addition, we analyzed the threshold ILE infusion rate that could lead to HTG.

## 2 Materials and methods

### 2.1 Study population

This study was a retrospective cohort study. We enrolled premature infants born at less than 32 weeks of gestational age (GA) who were admitted to the neonatal intensive care unit (NICU) at Ajou University Hospital between 2017 and 2022. Infants who underwent follow-up for TG levels within the first 10 days of life were included. The exclusion criteria were as follows: infants who died within 10 days of admission to the NICU; those for whom TG levels were not measured within the first 10 days of life; and those transferred from other hospitals with no medical records available prior to 10 days of life.

### 2.2 Nutrition protocol

Nutritional protocols and biochemical monitoring policies were implemented at our hospital in 2016 and revised in 2020. If all infants were capable of enteral nutrition (EN), trophic feeding was initiated, gradually increasing feeding with breast milk, premature infant formula, or hydrolyzed formula. For infants with a GA of less than 32 weeks, PN is initiated as a nutritional supplement starting from the first day of life if EN is insufficient (8). ILE administration begins at a dose of 0.5–1.0 g/kg/day and aims to reach 3–3.5 g/kg/day if tolerable by the end of the first week of life. If additional calories are required, the dosage may be increased to 4 g/kg/day (8). AA infusion starts at a dose of 2.0 g/kg/day and is increased by 0.5–1.0 g/kg/day based on daily urine output, the blood urea nitrogen, serum creatinine level observed in blood tests at 72 h and weekly. The glucose infusion rate (GIR) was also initiated at 5–6 mg/kg/min and gradually increased while monitoring blood glucose levels, ensuring that it did not exceed 12 mg/kg/min. If serum glucose levels rise above 200 mg/dl, adjustments should be made not only to the GIR but also to the dose of ILE. TG levels were monitored serially at 3, 7, and 10 days and weekly during PN administration. The ESPGHAN strongly recommends monitoring plasma TG levels in all infants, with a specific focus on the target threshold of 265 mg/dl (8). In contrast, the ASPEN also emphasizes the importance of TG monitoring by setting the threshold for HTG levels at 200 mg/dl (2, 3, 6). In this study, HTG was defined as the first serum TG value exceeding 200 mg/dl at any of these timepoints. To ensure temporal alignment between lipid exposure and HTG onset, the corresponding ILE dose and infusion rate were recorded on the same day that the elevated TG level was obtained. In HTG, ILE intake is reduced by approximately 0–2 g/kg/day based on the TG level. In this study, we define fat overload as administration rate exceeds the domestic approved rate of 0.125 g/kg/h based on SMOFLipid® (2).

### 2.3 Statistics

To analyze characteristics, *t*-tests were used for continuous variables, whereas chi-square or Fisher's exact tests were used for categorical variables. Continuous variables are expressed as the mean ± standard deviation. Logistic regression was used to identify the risk factors for HTG and examine the influence of HTG on neonatal diseases. In multivariate logistic analysis, we used a stepwise selection method to analyze the factors associated with HTG. To analyze the incidence of HTG, the mean levels of each TG, and the administered lipid intake according to GA and birth weight (BW), the chi-square and trend tests were employed. A receiver operating characteristic (ROC) curve was used to assess the lipid rate criteria for HTG. Statistical significance was set at *p* < 0.05.

## 3 Results

### 3.1 Characteristics

In total, 323 infants born before 32 weeks GA were enrolled in this study. Among them, 19 infants died within 10 days after birth, three infants were transferred with no medical records, and 25 infants had no recorded TG levels, resulting in the exclusion of these studies. The final study cohort comprised 276 infants. A total of 104 infants were diagnosed with HTG (37.7%), whereas the control group comprised 172 infants (62.3%) (Table 1). The mean GA of the HTG group was 27.3 ± 2.2 weeks, and the BW was 951.4 ± 239.8 g, which were significantly lower than that of the control group (28.8 weeks, 1,273.2 g, *p* < 0.001). The mean TG level in the HTG group was 287.8 mg/dl and when ILE dose administered 2.7 g/kg/day, which was significantly higher than that of the control group (2.0 g/kg/day, *p* < 0.001). Fat overload was observed more frequently in 34 infants in the HTG group (*p* < 0.001).

**Table 1:** The demographic characters of control and HTG group.

Variable	Control (*N* = 172)	HTG (*N* = 104)	*p*-value
Gestational age, weeks	28.9 ± 1.8	27.3 ± 2.2	<0.001
Birth weight, g	1,273.2 ± 313.3	951.4 ± 239.8	<0.001
1 min APGAR	4.32 ± 1.7	3.6 ± 1.7	<0.001
5 min APGAR	6.0 ± 1.6	5.2 ± 1.9	<0.001
Male, *n* (%)	96 (55.8)	55 (52.9)	0.727
Cesarean section, *n* (%)	151 (87.8)	83 (79.8)	0.106
SGA, *n* (%)	10 (5.8)	21 (20.2)	<0.001
Chorioamnionitis, *n* (%)	6 (3.5)	10 (9.6)	0.065
Preeclampsia, *n* (%)	37 (21.5)	27 (26.0)	0.483
GDM, *n* (%)	22 (12.8)	14 (13.5)	>0.999
Early sepsis, *n* (%)	4 (2.3)	3 (2.9)	>0.999
PDA, treated, *n* (%)	35 (20.4)	59 (56.7)	<0.001
RDS, *n* (%)	78 (45.4)	104 (100)	<0.001
Late sepsis, *n* (%)	9 (5.2)	14 (13.5)	0.03
NEC ≥ G2, *n* (%)	2 (1.2)	12 (11.5)	<0.001
IVH ≥ G3, *n* (%)	7 (4.1)	13 (12.5)	0.017
ROP ≥ stage 2, *n* (%)	9 (5.2)	21 (20.2)	<0.001
BPD, *n* (%)	59 (34.3)	87 (83.7)	<0.001
Mortality, *n* (%)	10 (5.8)	10 (9.6)	0.347
Serum TG, mg/dl	96.0 ± 42.0	287.8 ± 124.6	<0.001
Serum glucose, mg/dl	119.3 ± 38.6	157.4 ± 48.3	<0.001
Serum albumin, g/dl	3.3 ± 0.4	3.1 ± 0.4	<0.001
IV_glucose, mg/kg/min	10.1 ± 2.8	10.2 ± 3.0	0.914
IV amino acid, g/kg/day	2.3 ± 0.8	2.6 ± 0.8	0.005
IV lipid, g/kg/day	2.0 ± 1.0	2.7 ± 0.8	<0.001
Fat overload, *n* (%)	19 (11.1)	34 (32.7)	<0.001

HTG, hypertriglyceridemia; SGA, small for gestational age; GDM, gestational diabetes mellitus; PDA, patent ductus arteriosus; RDS, respiratory distress syndrome; NEC, necrotizing enterocolitis; IVH, intraventricular hemorrhage; ROP, retinopathy of prematurity; BPD, bronchopulmonary dysplasia; IV, intravenous.

Statistical significance was set at *p* < 0.05. Data presented as mean ± standard deviation or number (percentage).

**Figure 1:**
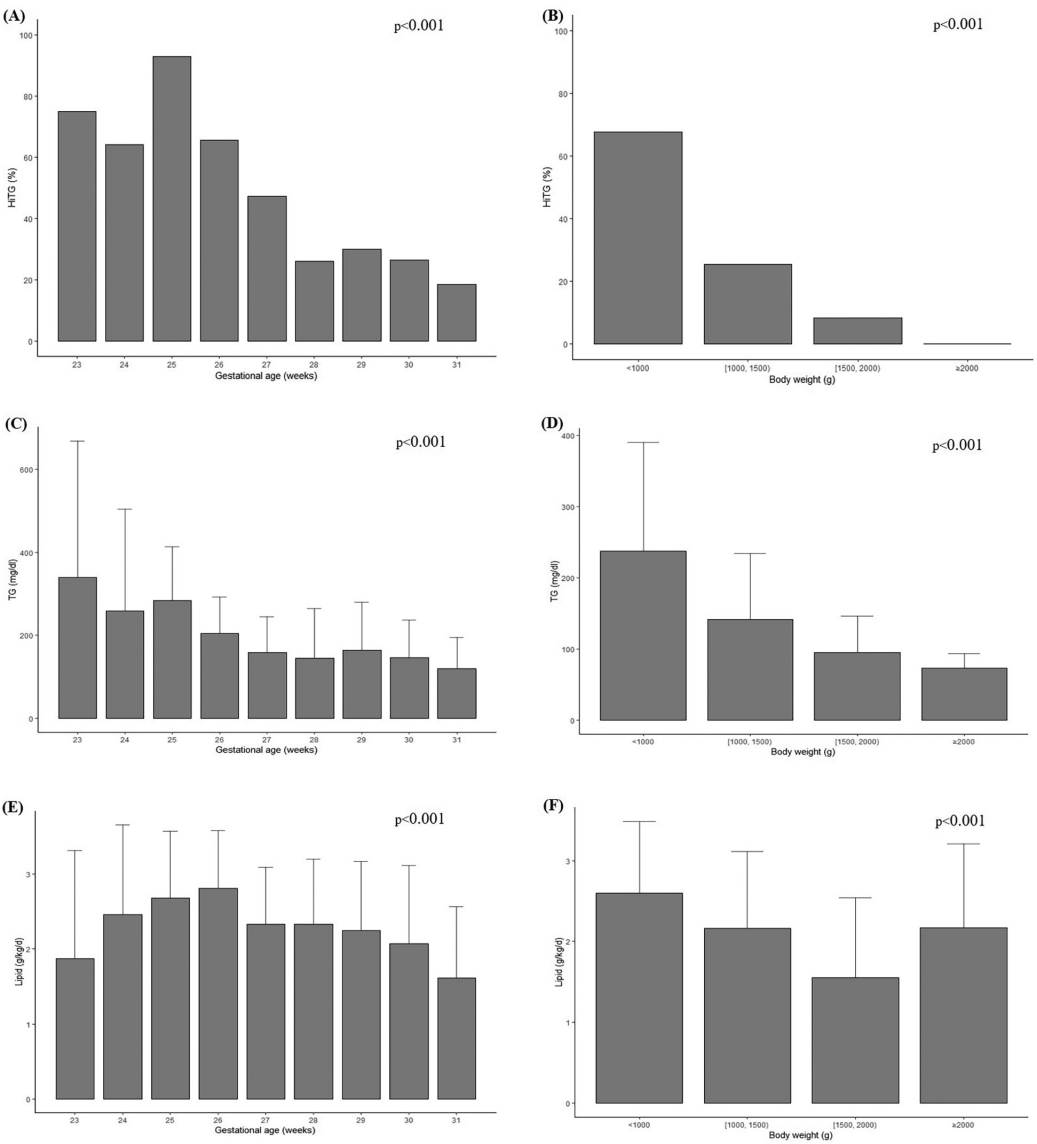
Incidence of HTG among GA **(A)**, among BW **(B)**, average serum TG level concentration among GA **(C)**, among BW **(D)**, administered IV lipid intake among GA **(E)**, among BW **(F)**. Chi square or trend test were used for analysis. Statistical significance was set at *p* < 0.05.

As shown in Figure 1, the analysis included the incidence of HTG, mean TG values, and average intravenous (IV) lipid dosage were analyzed according to GA and BW. The incidence of HTG tended to increase with lower GA, with the highest incidence observed at 25 weeks (92.9%) and the lowest at 31 weeks (18.6%) (*p* < 0.001) (Figure 1A). In addition, BW analysis revealed the highest proportion of HTG among infants weighing less than 1,000 g (67.7%) (*p* < 0.001) (Figure 1B). Similarly, the mean TG value peaked at 339.8 mg/dl at 23 weeks and 237.3 mg/dl in infants weighing less than 1,000 g (*p* < 0.001) (Figures 1C,D). A trend test was conducted to examine trends in TG levels and lipid dosages based on GA and BW. As a result, it was observed that the lower the GA and the smaller the BW, the higher the TG levels and the greater the lipid infusion amounts (*p* < 0.001) (Figures 1E,F).

### 3.2 Risk factors for HTG

Table 2 shows the analysis of the risk factors for HTG. Perinatal variables associated with HTG in the univariate analysis were GA, BW, 5 min APGAR score, small for gestational age (SGA), chorioamnionitis, patent ductus arteriosus (PDA), serum glucose level, IV AA intake, IV lipid intake, and fat overload. In multivariate logistic regression, BW (OR = 0.75; 95% CI, 0.66–0.85; *p* < 0.001), treated for PDA (OR = 2.47; 95% CI, 1.30–4.69; *p* = 0.006), IV lipid intake (OR = 1.79; 95% CI, 1.25–2.55; *p* = 0.001), and serum glucose level (OR = 1.01; 95% CI, 1.00–1.02; *p* = 0.023) were identified as final risk factors for HTG. However, fat overload did not appear to be the final risk factor in this analysis.

**Table 2:** Risk factors for HTG.

Variable	Univariable		*p*-value	Multivariable		*p*-value
	*β*	OR (95% CI)		*β*	Adjusted OR (95% CI)	
Gestational age	−0.37	0.69 (0.6, 0.78)	<0.001			
BW_100 g	−0.41	0.66 (0.59, 0.74)	<0.001	−0.29	0.75 (0.66, 0.85)	<0.001
5 min APGAR	−0.28	0.76 (0.65, 0.88)	<0.001			
Gender	−0.12	0.89 (0.55, 1.45)	0.636			
Delivery	−0.6	0.55 (0.28, 1.07)	0.076			
SGA	1.41	4.1 (1.85, 9.11)	<0.001			
Chorioamnionitis	1.08	2.94 (1.04, 8.35)	0.043			
Preeclampsia	0.25	1.28 (0.72, 2.26)	0.397			
GDM	0.06	1.06 (0.52, 2.18)	0.873	0.92	2.51 (0.98, 6.43)	0.056
Early sepsis	0.22	1.25 (0.27, 5.69)	0.775			
PDA	1.64	5.13 (3, 8.78)	<0.001	0.9	2.47 (1.3, 4.69)	0.006
Glucose	0.02	1.02 (1.01, 1.03)	<0.001	0.01	1.01 (1, 1.02)	0.023
IV lipid	0.83	2.28 (1.69, 3.09)	<0.001	0.58	1.79 (1.25, 2.55)	0.001
IV amino acid	0.45	1.57 (1.14, 2.17)	0.006			
Fat overload	1.36	3.91 (2.09, 7.33)	<0.001			

HTG, hypertriglyceridemia; BW_100 g, birth weight divided by 100 g; SGA, small for gestational age; GDM, gestational diabetes mellitus; PDA, patent ductus arteriosus; IV, intravenous.

Statistical significance was set at *p < 0.05.*

### 3.3 Time to normalize TG levels

In this study, we examined whether reducing the IV lipid intake when HTG occurs is associated with a shorter time for TG levels to normalize. The average time taken for normalization of TG levels was 4.9 ± 5.0 days when reducing ILE doses by 0.27 ± 0.46 g/kg/day. The time taken for TG levels to normalize was typically between 1 and 3.7 days, accounting for approximately 56% of the cases. In linear regression, correcting the amount of lipid reduction was positively associated with both the TG levels within the first 10 days and the number of days taken for TG normalization (*β* = 0.009) (*p* = 0.024).

Logistic regression analysis was conducted to investigate whether rapid normalization of HTG was associated with reduced rates of late sepsis, necrotizing enterocolitis (NEC) grade 2 or higher, intraventricular hemorrhage (IVH) grade 3 or higher, retinopathy of prematurity (ROP) stage 2 or higher, bronchopulmonary dysplasia (BPD), and mortality. Statistically significant findings confirmed that only ROP stage 2 or higher, indicated a decreased risk associated with faster normalization of TG, in accordance with our research (OR = 0.59; 95% CI, 0.40–0.88; *p* = 0.011) (Table 3).

**Table 3:** Multivariable logistic analysis between time taken for normalization of TG and neonatal morbidities.

Morbidities	*β*	Adjusted OR (95% CI)	*p*-value
Late sepsis	−0.03	0.97 (0.82, 1.14)	0.711
NEC G2 or higher	−0.1	0.9 (0.76, 1.08)	0.255
IVH G3 or higher	−0.09	0.91 (0.78, 1.06)	0.215
ROP stage 2 or higher	−0.52	0.59 (0.40, 0.88)	0.011
BPD	−0.05	0.95 (0.86, 1.05)	0.278
Mortality	0.04	1.04 (0.89, 1.22)	0.631

TG, triglyceride; NEC, necrotizing enterocolitis; IVH, intraventricular hemorrhage; ROP, retinopathy of prematurity; BPD, bronchopulmonary dysplasia.

Statistical significance was set at *p* < 0.05.

### 3.4 Fat overload–analysis of ILE infusion rate that can cause HTG

**Figure 2:**
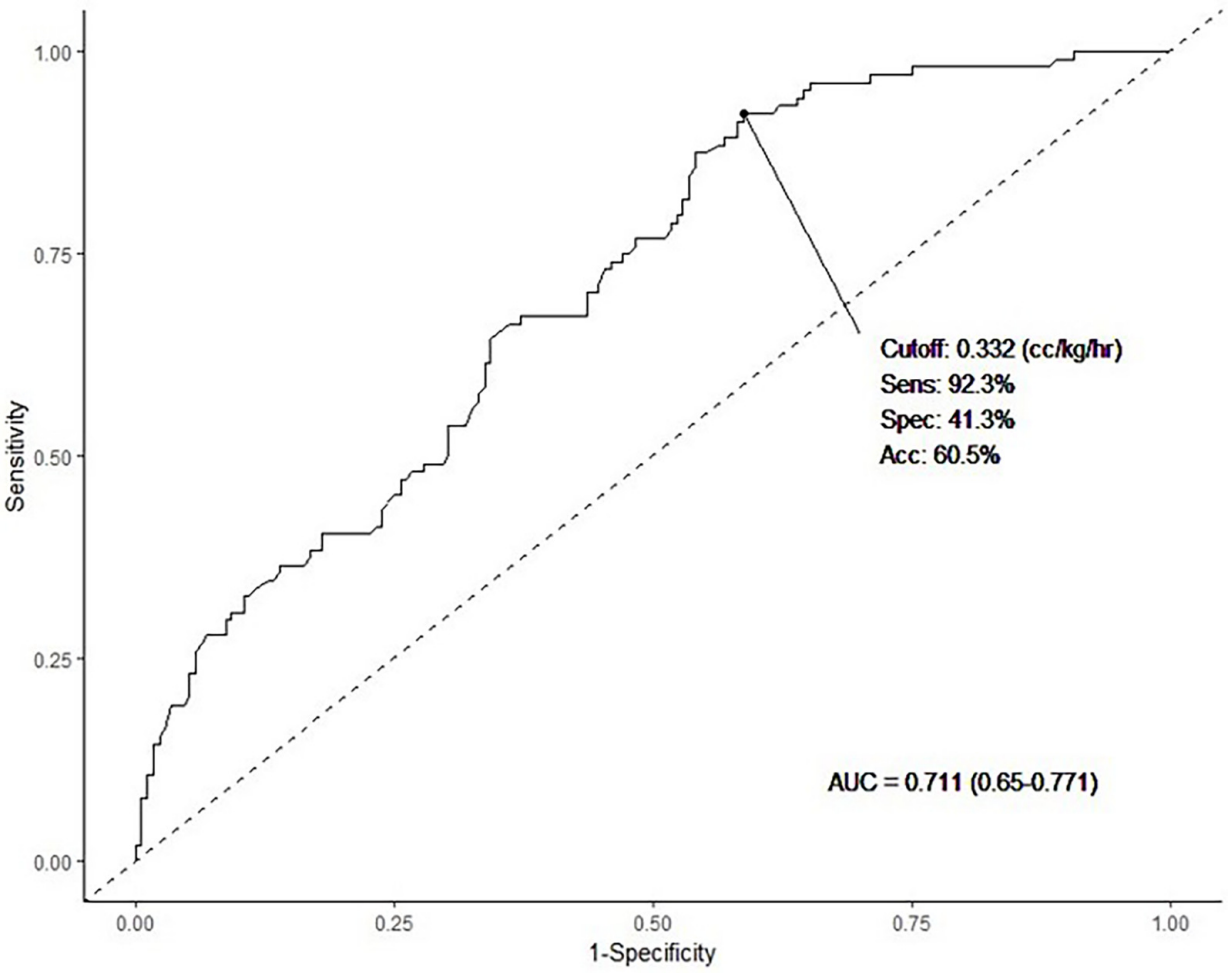
We investigated the predicted lipid infusion rate for potential development of HTG using ROC curve analysis. The cutoff value was 0.332 cc/kg/h (1.6 g/kg), sensitivity was 92.3%, specificity was 41.3%, and the AUC was 0.711 (*p* < 0.001). Statistical significance was set at *p* < 0.05.

When comparing the ILE infusion rates between the control group and HTG group, it was observed that the rates were 0.39 cc/kg/h (2 g/kg/day) and 0.54 cc/kg/h (2.6 g/kg/day), respectively. This indicated that the infusion rate was higher in the HTG group (*p* < 0.001). In this study, we aimed to determine the cutoff value of the ILE infusion rate that induced HTG in the HTG group. In Figure 2, the ROC curve is presented, showing that the cutoff value for fat overload in the HTG group was 0.332 cc/kg/h (1.6 g/kg/day). The sensitivity was relatively high (92.3%), and the AUC was 0.711, indicating fair accuracy.

## 4 Discussion

In preterm infants, ILE administration is a routine practice that supports caloric intake in those who do not receive sufficient EN. In this study, we analyzed the incidence and risk factors of HTG as well as the effects of ILE reduction and fat overload in preterm infants born before 32 weeks of gestation, following our nutrition guidelines.

HTG occurred in 37.7% of cases, which is higher than previous reported values (19.5%–29.7%) (1, 4, 13). Differences in diagnostic thresholds (e.g., 250 mg/dl vs. 200 mg/dl) and the inclusion of more immature and smaller infants in our study likely contributed to this discrepancy (1, 4, 13). Our cohort included infants with a mean GA of 27.3 ± 2.2 weeks and BW of 951.4 ± 239.8 g, lower than prior studies. The inclusion of a greater number of smaller and more premature infants in our study likely contributed to the higher incidence of HTG.

Consistent with existing research, BW was a significant risk factor for HTG, while GA and SGA were not (4, 6, 13, 14). This suggests that BW may better reflect nutritional vulnerability than GA alone. Our findings are supported by studies reporting that lower BW increases the risk of HTG, particularly in the early neonatal period when PN is heavily relied upon (5, 13). Reliance on PN may be further prolonged in cases requiring PDA treatment, due to delayed progression of EN (5). Previous studies have shown that early lipid infusion is not associated with an increased incidence of PDA (15–17). In cases where symptomatic PDA was treated with medication or ligation, the amount of enteral feeding likely decreased owing to the surgical conditions and side effects of the general condition. This likely increased their reliance on PN, a consideration supported by previous studies that reported similar results (6). Serum glucose levels were found to be significantly associated with HTG in our multivariate analysis, although the odds ratio was modest (OR = 1.01; 95% CI, 1.00–1.02). This may reflect complex metabolic interactions rather than a direct causal relationship. For example, the administration of ILE can increase free fatty acid levels, leading to reduced insulin sensitivity in peripheral tissues, impaired glycolysis, and increased gluconeogenesis—all of which may contribute to hyperglycemia in preterm infants (15, 18, 19). Furthermore, hyperglycemia in extremely low birth weight (ELBW) infants is common even in the absence of lipid infusion, due to factors such as stress, infections, steroid use, and insulin resistance (15, 20). Thus, while serum glucose level may serve as a marker of metabolic dysregulation associated with HTG, additional studies are needed to delineate the direction and mechanism of this association.

In this study, we analyzed the incidences of HTG, serum TG levels, and ILE infusion amounts based on GA and BW. A clear inverse trend was observed, where lower GA and BW were associated with higher HTG incidence, elevated TG levels, and greater lipid infusion doses. This reflects the limited enteral tolerance and higher dependence on PN among smaller and more immature infants. An exception to the trend was noted in the >2,000 g subgroup in Figure 1F, which showed a sudden increase in lipid dose. This is likely attributable to the small number of infants in this category and may not represent a true deviation. Interestingly, previous studies have reported an increasing trend in ILE dose with advancing GA (6). This discrepancy can be explained by the fact that in more mature infants, EN is typically established earlier, leading to a natural reduction in PN volume. In contrast, many ELBW infants in our cohort likely remained on PN during the first month due to feeding intolerance, resulting in higher cumulative lipid exposure.

In this study, with the exception of mortality, the incidence of other neonatal morbidities was higher in the HTG group (Table 1). Previous studies have investigated whether HTG can predict morbidity, and multivariate analysis revealed no significant association (4, 21, 22). However, other reports have indicated that HTG is a risk factor for ROP at low GA, specifically between 23 and 25 weeks (1). We investigated whether the time to TG normalization was associated with any neonatal morbidities and found a statistically significant association only with ROP stage 2 or higher. Docosahexaenoic acid (DHA) is an omega-3 fatty acid synthesized from alpha-linolenic acid, plays a crucial role in the development of the brain and retina (15, 23). Consequently, many studies have focused on the importance of early introduction of ILE in VLBW and ELBW infants (15). While Douglas et al. observed that the absence of ILE was associated with a higher risk of ROP (7), other reports did not find any significant difference in the ROP incidence between early and delayed ILE groups (15, 17, 24, 25). Given that ROP incidence is strongly associated with lower GA and BW, the observed association between HTG and ROP in our study could be influenced by these confounding factors. However, the persistence of the association with TG normalization time raises the possibility of a more direct metabolic link. One potential mechanism involves arachidonic acid metabolism. Arachidonic acid, an essential fatty acid in lipid metabolism, is a precursor to thromboxane—a compound produced via the cyclooxygenase pathway (26). ILE administration may alter this pathway and increase thromboxane levels, which can lead to retinal vasoconstriction, hypoxia, and reactive neovascularization, all of which are implicated in the pathogenesis of ROP (16, 26). Therefore, it is plausible that the faster normalization of TG levels in infants with HTG could mitigate excessive lipid metabolism and thromboxane production, thereby reducing the risk of ROP. While causality cannot be confirmed in our observational study, these findings suggest a potential biological link between lipid metabolism and retinal vascular pathology that merits further investigation.

We also examined whether reducing ILE dose could shorten the time to TG normalization. Although there was a negative correlation between the amount of lipid reduction and the number of days required for TG levels to normalize, this relationship was not statistically significant (*β* = −0.612; *p* = 0.556). Notably, higher TG levels were significantly associated with longer normalization times, suggesting that the severity of HTG itself is an important factor. This supports the clinical need to reduce the ILE dose when HTG occurs to facilitate recovery.

Finally, we determined the cutoff value for the ILE administration rate for all premature infants included in this study. This value was 0.332 cc/kg/h (1.6 g/kg/day), which was significantly lower than the domestic approval rate. With a sensitivity of 92.3% and an AUC of 0.711, this threshold may indicate an increased risk for HTG when exceeded. However, given the relatively low specificity (41.3%), this cutoff should not be interpreted as an absolute threshold for clinical decision making. Instead, it may serve as a reference point to highlight the importance of careful and individualized monitoring, particularly in preterm infants with lower birth weight or multiple risk factors. Our findings align with the ASPEN recommended dosing range of 1–3 g/kg/day, but suggest that HTG may still occur within this range, emphasizing the need for serum TG monitoring even when standard dosing is applied. As various factors influence HTG in preterm infants, caution is advised when approaching the upper limits of ILE infusion. To ensure adequate caloric support without inducing metabolic complications, lipid administration should be balanced with close TG monitoring and individualized risk assessment.

This retrospective study was subject to potential selection and reporting biases. Additionally, as this was a single-center study, it was limited by its small sample size. Another limitation is that while overall guidelines were followed for adjusting lipid administration, variations in the amounts adjusted by different individuals could introduce inconsistencies. However, this study had several strengths. It is the first to analyze the time required for HTG normalization while adjusting lipid administration and to examine the impact of faster normalization on neonatal morbidities. Additionally, this is the first study to analyze the critical ILE administration rate that can prevent fat overload syndrome, thereby providing valuable insights for clinical practice.

In conclusion, HTG is a common complication observed in premature infants. Lower BW, PDA, serum glucose levels, and higher ILE dosage were associated with increased HTG risk. Moreover, these findings suggest that while exceeding a certain lipid infusion rate may increase the risk of HTG, this value should not be interpreted as a fixed clinical threshold. Instead, it highlights the importance of individualized assessment and vigilant monitoring. Our results emphasize the need for customized and carefully titrated nutritional strategies to optimize growth while minimizing the metabolic risk in this vulnerable population. Furthermore, establishing precise guidelines for titrating ILE during HTG is necessary.

## Data Availability

The original contributions presented in the study are included in the article/Supplementary Material, further inquiries can be directed to the corresponding author.
